# Suspected chest malignancy hiding *Aggregatibacter actinomycetemcomitans* lung infection – case report and review of literature

**DOI:** 10.3389/fped.2026.1801660

**Published:** 2026-04-29

**Authors:** Ariane Gérard, Anna-Maria Charatsi, Sophie Huybrechts, Luis Perez Casanova, Chantal Tsobo Blistain, Paul Philippe, Sébastien Mesureur, Jean Paul Van Nieuwenhuyse, Isabel de la Fuente Garcia

**Affiliations:** 1 National Pediatric Center, Centre Hospitalier de Luxembourg (CHL), Luxembourg City, Luxembourg; 2 National Pathology Center, Laboratoire National de Santé (LNS), Dudelange, Luxembourg; 3 Microbiology Department, Centre Hospitaliser de Luxembourg (CHL), Luxembourg City, Luxembourg; 4 National Pediatric Surgery Center, Centre Hospitalier de Luxembourg (CHL), Luxembourg City, Luxembourg; 5 Pediatric Radiology, Centre Hospitalier de Luxembourg (CHL), Luxembourg City, Luxembourg; 6 Faculty of Science Technology and Medecine, University of Luxembourg, Esch, Luxembourg

**Keywords:** *Aggregatibacter actinomycetemcomitans*, case report, empyema necessitans, lung infection, pediatric infectious disease

## Abstract

Empyema necessitans is a rare complication of parapneumonic effusion. Here, we present the case of an eight-year-old girl who presented with a tumor-like chest mass. The clinical presentation initially suggested a malignant disease. A multidisciplinary approach prior to surgery enabled a correct diagnosis by performing pre-surgery cultures and submitting surgical specimens for both microbiological and histopathological analysis. The microbiological workup demonstrated an *Aggregatibacter actinomycetemcomitans* infection, which was consistent with the histopathological findings. Antibiotic therapy was administered for a total of 8 weeks, leading to the complete resolution of the symptoms and the lesion. This article provides a detailed description of this exceptional infection in children that can initially be misdiagnosed as a malignant lesion and offers a review of the limited literature on published cases in pediatrics.

## Introduction

Empyema necessitans, also known as necessitatis, is a type of pleural empyema that spreads into the soft tissue of the chest wall. It is a rare complication of infectious pleuropneumonia, particularly in children. *Aggregatibacter actinomycetemcomitans* (formerly *Actinobacillus actinomycetemcomitans*), a fastidious Gram-negative bacterium from the oral microbiota, can cause various human infections (1–3). Although several cases of empyema necessitans caused by this bacterium have been reported, occurrences in children are rare.

## Case description

An 8-year-old girl with no previous medical history was referred by her General Practitioner to the Pediatric Emergency Department for a right parasternal mass and abnormal blood analysis (Hemoglobin at 7.0 g/dl, Platelets at 700,000/mm3). The mass was noticed only 3 days before the visit. The girl was weak, sleepy, and had no appetite for a week, along with night sweats for two days. She lost 8 kg over the last two months, and had initially no fever, cough, or other symptoms. She had no history of recent dental infection or oral symptoms before admission. On examination, she had a sensitive right parasternal mass without redness and small cervical and inguinal adenopathies. Cardio-pulmonary auscultation and abdominal examination were unremarkable.

**Figure 1:**
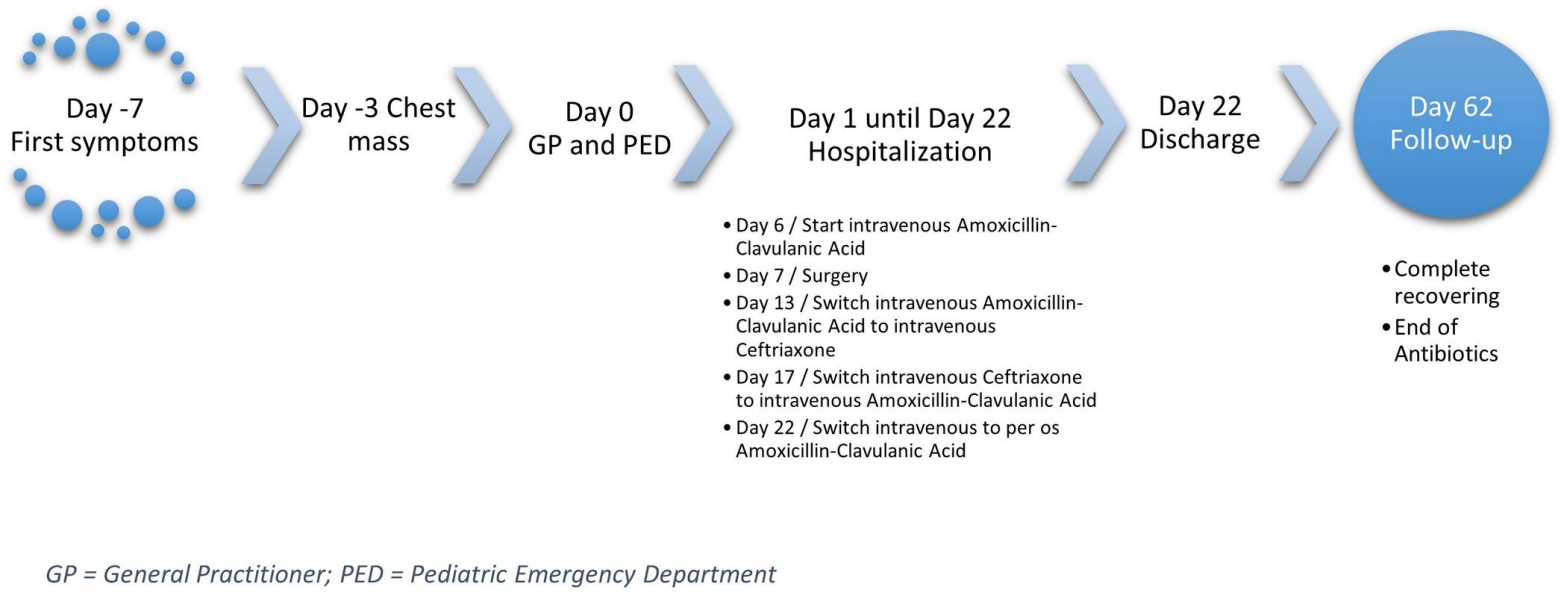
Flowchart showing the major steps in disease development and treatment.

**Figure 2:**
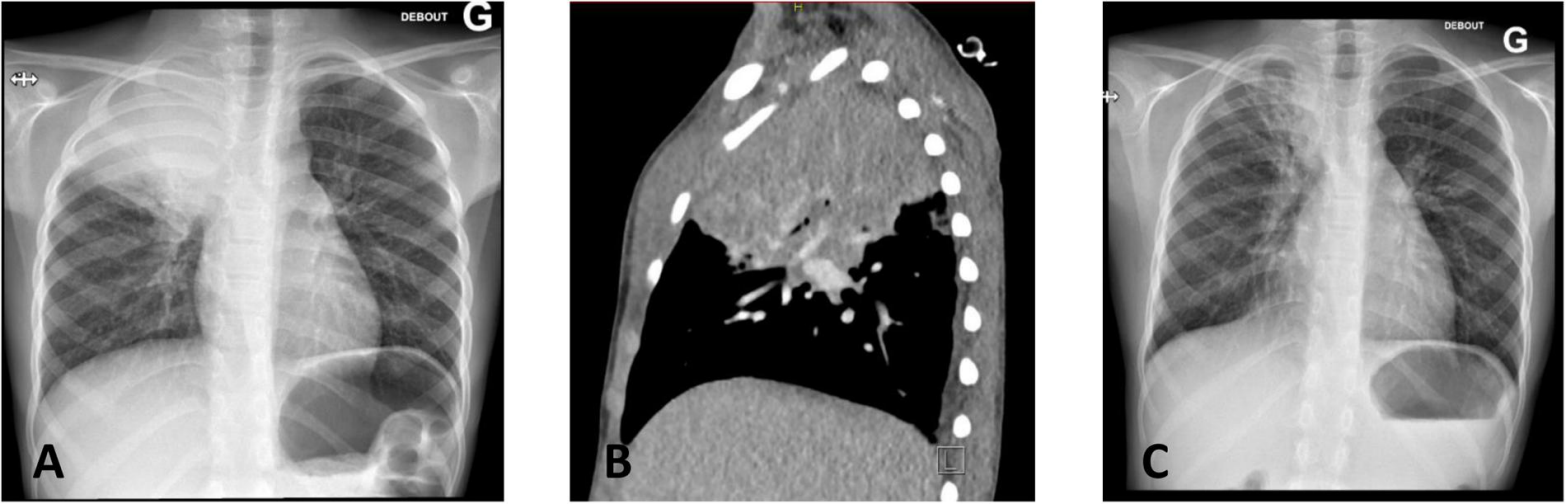
Radiological findings of our patient: **(A)** first X-ray showing consolidation of the right upper lobe – **(B)** computed tomography before treatment showing consolidation of the right upper lobe with extension in the anterior chest wall – **(C)** X-ray after treatment completion showing complete regression of the consolidation.

Hospitalization followed for further tests (see Figure 1 - Timeline). Blood samples showed a moderate inflammatory syndrome (C-Reactive Protein at 76 mg/L), neutrophilic hyperleukocytosis (leukocytes at 14,360/mm^3^, neutrophils at 9,790/mm^3^), microcytic anemia (hemoglobin 8.7 g/dl, MCV 69.2fl), and thrombocytosis (platelets at 764,000/mm^3^). Chest X-ray showed a right apical opacity with an aerated bronchogram (Figure 2A). Ultrasound revealed fleshy tissue between muscles extending through an intercostal space to an intrathoracic mass. A thoracic CT confirmed right upper lobe condensation with invasion of the submuscular fatty structure (Figure 2B). An abnormality of the neck vessels was noted, with the right venous brachiocephalic trunk and right subclavian vein not opacified.

Tests for tuberculosis were negative (QuantiFERON, intradermal reaction and microbiological testing of respiratory specimens). Aspergillosis was ruled out with a negative Galactomannan test. Blood flow cytometry showed predominantly T-cell lymphopenia, and no blasts. 5FDG PET-CT showed intense hypermetabolism in the right lung condensation and adjacent tissues, with no other hypermetabolic focus.

### Diagnostic assessment

Differential diagnosis included in the first place a neoplastic etiology or an infectious pathology, particularly tuberculosis. Due to weight loss, hypermetabolic mass on PET-FDG imaging, and tissue invasion, neoplastic pathology was initially suspected, prompting a thoracoscopy on day 7 for diagnosis and treatment. A thorough evaluation of airway and vascular compression should be performed in patients with thoracic masses before general anaesthesia to prevent any complications.

**Figure 3:**
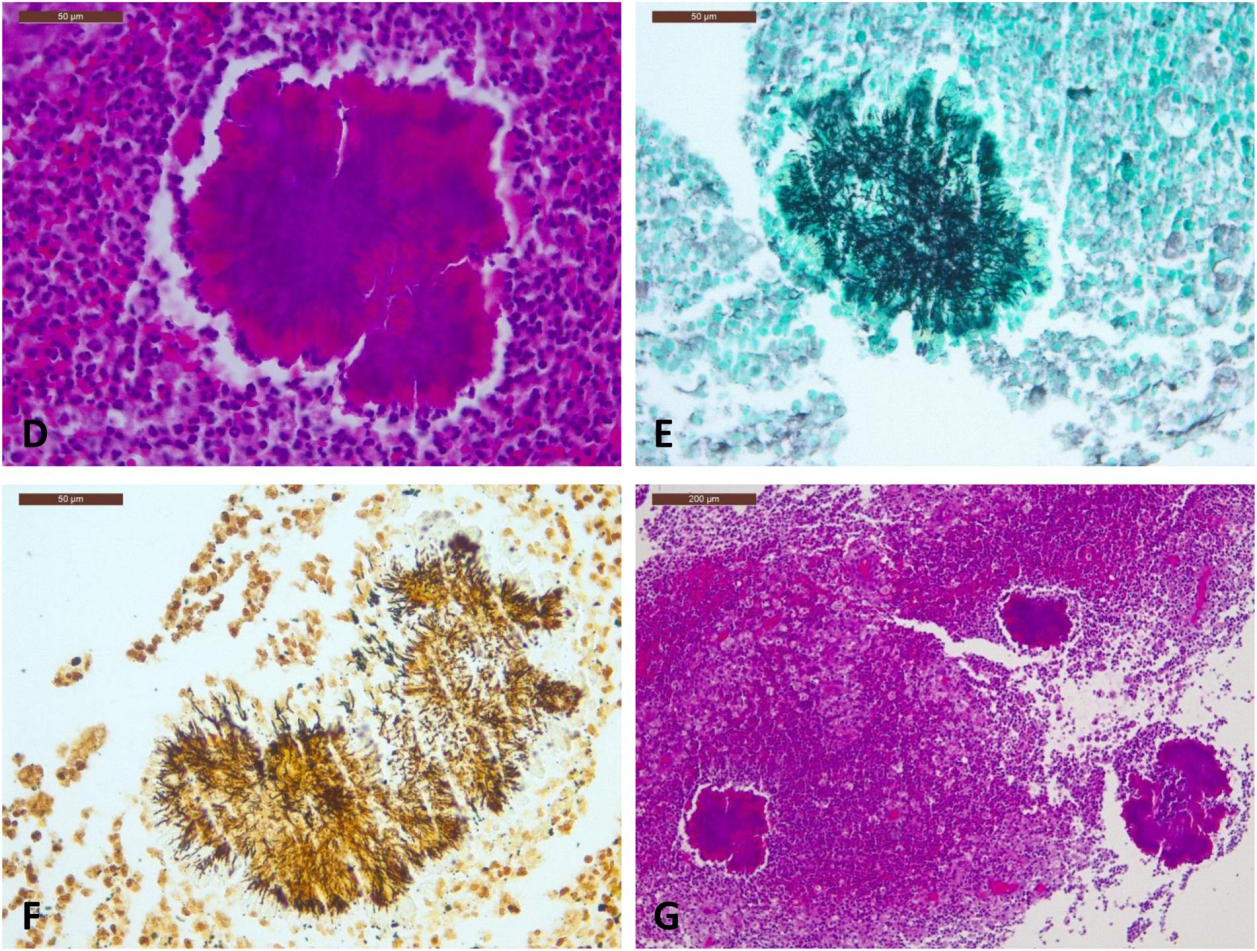
Histopathological findings of our patient suggestive of actinomycosis: **(D)** hematoxylin-eosin staining, magnification ×40, demonstrating sulfur granules composed of filamentous bacterial colonies- **(E)** the same bacterial colonies stained with grocott methenamine silver, magnification × 40 – **(F)** the same bacterial colonies stained with warthin-starry, magnification × 40 – **(G)** hematoxylin-eosin staining, magnification ×10, demonstrating inflammatory exudate rich in neutrophils and histiocytes, with multiple basophilic sulfur granules.

Histological examination of biopsies taken from the pleura, right upper lung lobe, and anterior and posterior thoracic walls revealed a dense acute-on-chronic inflammatory infiltrate, composed of neutrophils, lymphocytes, plasma cells, and histiocytes, associated with fibrosis and focal skeletal muscle fiber atrophy. There was no significant cytological atypia or evidence of malignancy. In the anterior wall biopsy, multiple basophilic sulfure granules were identified within inflammatory cells. These structures consisted of radiating filamenous bacterial colonies with peripheral eosinophilic club-like material, consistent with Splendore-Hoeppli phenomenon (Figure 3D, G). These bacterial colonies were highlighted by Grocott methenamine silver (Figure 3E) and Warthin-Starry stains (Figure 3F). Overall, the histopathological features were highly suggestive of thoracic Actinomycosis. Differential diagnostic considerations included Nocardia; however, the absence of branching delicate beaded filaments and the histomorphologic appearance of compact sulfur granules favored Actinomyces. Correlation with microbiological analysis was recommended for confirmation.

Cultures of samples taken during the procedure were performed on different media, with incubation lasting up to seven days. Microscopic examination showed numerous leukocytes but no microorganisms. Aerobic culture on COS^1^ medium and anaerobic culture on PVX^2^ medium, both incubated in a CO_2_-enriched atmosphere (5% +/- 2% CO_2_), revealed a bacterial colony, which was subsequently identified by mass spectrometry as *A. actinomycetemcomitans*. Antibiotic susceptibility testing performed according to EUCAST criteria on PVX medium showed that the organism was resistant to Amoxicillin and Ampicillin but sensitive to Amoxicillin-Clavulanic Acid. The identification of *A.actinomycetemcomitans* was further confirmed through 16S rRNA PCR.

### Evolution

Before starting antibiotics, blood tests showed increasing inflammation and neutrophilic hyperleukocytosis (C-Reactive Protein 137 mg/L; sedimentation rate 120 mm/h). She developed fever during hospitalization. The blood culture was sterile. Echocardiography on day 8 showed no evidence of endocarditis despite the presence of a suspect germ (*A. actinomycetemcomitans* being part of the HACEK group).

Intravenous Augmentin at a dose of 100 mg/kg/day was started on day 6 of hospitalization initially as surgical prophylaxis and continued for 7 days. Intravenous Ceftriaxone 2 g/day was started on day 13 after initial thoracoscopic culture results became available. These were positive for a Gram-negative bacterium, later identified as *A. actinomycetemcomitans,* and the treatment was continued for 4 days. Based on the antibiogram, intravenous Augmentin 100 mg/kg/day was resumed on day 17 for 5 days, resulting in a total of 16 days of intravenous antibiotics. The patient improved clinically and biologically, with CRP dropping to 9.5 mg/L at discharge. Oral Augmentin at a dose of 60 mg/kg/day was set up for a total antibiotic therapy of 2 months.

During hospitalization, she required three red blood cell transfusions and folic acid for microcytic anemia, likely secondary to inflammation and blood loss during thoracoscopy. She also received intravenous hydration, and prophylactic Clexane for blood flow issues in the right deep cervical veins. Anti-coagulant treatment was stopped at discharge. A follow-up ultrasound 15 days later showed normalized blood flow.

After discharge, dental care addressed the infection source. She had had poor dental hygiene and had never been followed by a dentist.

Six weeks post-discharge, the patient had gained 4 kg, with no recurrence of fever or respiratory symptoms. The mass disappeared, and cardiopulmonary auscultation remained normal. A chest X-ray showed reduced opacity, and CRP was 0.51 mg/L. Antibiotics were stopped due to favorable clinical, laboratory and radiological improvement. A follow-up examination 6 months later confirmed full recovery (Figure 1C).

## Discussion and literature review

Empyema necessitans, a rare complication of pleuropneumonia, involves infection spreading into the chest wall (1). It is particularly uncommon in children (2) and can be caused by various pathogens, including *Streptococcus pneumoniae*, *Mycobacterium tuberculosis*, *Staphylococcus aureus*, *Streptococcus Milleri*, and *Actinomycosis* (2, 3). Our patient, presenting with a chest mass, weight loss, and night sweats, was diagnosed with empyema necessitans caused by *A. actinomycetemcomitans*. Despite these symptoms suggestive of malignancy, an infectious diagnosis was confirmed. Multidisciplinary discussions are crucial before surgery in order to collect appropriate samples for both infectious (using appropriate culture media) and neoplastic hypotheses. Table 1 shows major chest malignancies in children and their clinical, radiological and pathological characteristics in comparison with Empyema necessitans.

**Table 1:** Clinical presentation, radiological characteristics and pathological features of relevant malignant differential diagnoses in comparison to empyema necessitans, based on dillman et al. (11), Zapala et al. (12), Chetaille et al. (13) and Cancérologie de l'enfant (14).

Pathology	Clinical presentation	Radiological characteristics	Pathological Features
Rhabdomyosarcoma	Chest wall mass, initially not painful Adenopathies may be present	A soft tissue tumor of the chest wall with occasionally aggressive features like bone erosion	Features of immature skeletal muscle differentiation
Ewing Sarcoma	Painful chest wall mass Fever, weight loss, altered general condition	A tumor typically arising from bone and more often osteolytic, commonly associated with periosteal reactions, frequent involvement of soft tissue	Small round blue cells, possible necrotic areas
Osteosarcoma	Painful chest wall mass (More often seen in long bones)	A bone tumor with variable imaging appearance (osteolytic, sclerotic or mixed)	Atypical osteoblasts with osteoid matrice
Synovial sarcoma	Chest wall mass	A well-defined tumor with heterogeneous postcontrast enhancement, sometimes cystic or with calcifications	Spindle cells, sometimes associated with epithelial cells
Other sarcomas	Chest wall mass Extremely rare in pediatrics	Depending on the type of sarcoma	Depending on the type of sarcoma
Neuroblastoma	Rarely palpable chest mass Cough, dyspnea Opsoclonus-myoclonus Fever, altered general condition Pain in multiple localizations	Mostly paravertebral mass, sometimes with calcifications	Small round blue cells with Schwannian stromal cells
Lymphomas * Lymphoblastic T * Hodgkin	No palpable chest mass Multiple adenopathies, hepatomegaly/splenomegaly Fever, weight loss, altered general condition, night sweats Cough, shortness of breath, chest pain	Mediastinal soft tissue mass Multiples adenopathies Sometimes pulmonary involvement	* T lymphoblasts * Hodgkin cells
Germ cell tumors	No palpable chest mass Sometimes asymptomatic Chest pain, cough, dyspnea, superior vena cava syndrome Fever, weight loss, altered general condition	Mostly anterior mediastinal mass, irregular margin Variable components: soft tissue, fat, calcification, cyst, necrosis and others	Depending on the type of germ cell tumors
Empyema necessitans	Thoracic mass, sometimes painful Fever, altered general condition, weight loss Cough	Primary pulmonary origin with extension into the soft tissue of the chest wall	Inflammatory pattern No cytological atypia

*A. actinomycetemcomitans* is a slow-growing, capnophilic, facultatively anaerobic, small gram negative rod first described in 1912 and reclassified in 2006 (4). This Bacterium is part of the oral flora but can cause various infections, often in association with *Actinomyces*. Delays in diagnosis are common, ranging from days to over a year (2, 5). *A. actinomycetemcomitans* is primarily associated with periodontal disease, particularly localized juvenile periodontitis (6, 7). However, it can also cause other localized or systemic infections (8), including endocarditis (as part of the HACEK group) (9), brain abscesses, pneumonia, and osteoarticular infections (5, 10). Improved oral hygiene may help reduce the risk of such infections.

In our case - a rare pediatric instance of empyema necessitans caused by *A. actinomycetemcomitans* - the initial presentation suggested a neoplastic pathology. The infectious nature of the mass was confirmed through surgical sample and culture. Poor dental hygiene was identified as the source of the infection.

A review of the literature on PubMed and Embase in December 2025 using the search terms ‘empyema necessitans’ OR ‘empyema necessitatis’ OR ‘chest wall infection’ OR ‘thoracic wall infection’ OR ‘thoracic infection’ AND (*Aggregatibacter actinomycetemcomitans* OR *Actinobacillus actinomycetemcomitans*) identified twelve other pediatric cases of empyema necessitans caused by this bacterium alone or in combination with other pathogens (see Table 2). Diagnosis typically involved cultures from surgical samples. Penicillin, often with Clavulanic Acid, was the common treatment, initially administered intravenously, followed by an oral course lasting up to six months. As in most of the reported cases, our patient did not require surgical drainage as treatment. The antibiotic therapy we used (for reference: Ceftriaxone and Amoxicillin-Clavulanic Acid) was similar to that used in other reported cases but of shorter duration. All patients, including ours, showed good or full recovery.

**Table 2:** Cases of pediatric empyema necessitans caused by A. actinomycetemcomitans: patient demographics, treatments and outcomes based on literature review.

Case N°	Author and date	Age	Sex	Microbiology	Antibiotics, administration, and durations	Surgical drainage	Outcome
**1**	Kaplan et al. 1971 (5)	10y	Male	*A. actinomycetemcomitans*	Unkown antibiotic oral 3 months + Penicillin G IV for 1 month + Penicillin VK oral for 1 year	No	Good recovery
**2**	Carlile et al. 1983 (15)	13y	Male	*A. actinomycetemcomitans*	Ampicillin 5 weeks	No	Full recovery
**3**	Chen et al. 1995 (16)	14y	Female	*A. actinomycetemcomitans*	Cefoxitin IV 2 weeks + Amoxicillin oral for a total of 3 months	Yes	Full recovery
**4**	Hagawira et al. 2009 (17)	9y	Female	*A. actinomycetemcomitans*	Cefotaxim IV and Azithromycin oral for 10 days + Amoxicillin oral for 1 year	Yes	Full recovery
**5**	Nash et al. 2014 (18)	11y	Male	*A. actinomycetemcomitans*	Penicillin IV for 6 days + Ampicillin-Sulbactam IV for 8 weeks + Amoxicillin-Clavulanic Acid oral for 16 weeks	No	Full recovery
**6**	Moskowitz et al. 2015 (19)	8y	Female	*A. actinomycetemcomitans + Actinomyces israelii*	Imipenem IV 5 weeks + Ceftriaxone IV + Doxycycline oral for a total of 1 year	Yes	Full recovery
**7**	Shilo et al. 2015 (20)	11y	Male	*A. actinomycetemcomitans*	Penicillin G IV for 2 months + Amoxicillin oral and Clindamycin oral for 10 months	No	Full recovery
**8**	Shilo et al. 2015 (20)	14y	Male	*A. actinomycetemcomitans*	Ampicillin-Sulbactam IV for 18 days + Amoxicillin-Clavulanic Acid oral and Amoxicillin oral for at least 3 months	Yes	Good recovery
**9**	Robbins et al. 2019 (21)	11y	Female	*A. actinomycetemcomitans*	Ceftriaxone IV for 8 weeks + Amoxicillin oral for 4 weeks	No	Full recovery
**10**	Marquez et al. 2020 (2)	17y	Female	*A. actinomycetemcomitans + Fusobacterium nucleatum + Eikenella corrodens + Actinomyces israelii*	Amoxicillin-Clavulanic Acid oral for 6 months	No	Full recovery
**11**	Benhaïm-Mattout et al. 2022 (10)	7y	Female	*A. actinomycetemcomitans*	Amoxicillin oral for 2 months	No	Good recovery
**12**	Samarro et al. 2024 (22)	9y	Female	*A. Actinomycetemcomitans*	Cephalexin oral 10 days + Sulfamethoxazole-Trimethorprim oral + Cefotaxime IV for 28 days + Amoxicillin-Clavulanic Acid oral for 8 months	No	Good recovery
**13**	Our case	8y	Female	*A. actinomycetemcomitans*	Amoxicillin-Clavulanic acid IV for 7 days + Ceftriaxone IV for 9 days + Amoxicillin-Clavulanic Acid oral for 6 weeks	No	Full recovery

IV: intravenously.

In the absence of specific guidelines for this infection, the duration of antibiotic therapy should be primarily guided by the patient's clinical evolution. Surgical drainage is sometimes performed in addition to antibiotic therapy. Our approach, consistent with the literature, involved intravenous antibiotics followed by oral therapy. The treatment lasted eight weeks, which is shorter than in most reported cases (often lasting more than two months), and there was no recurrence. We would suggest that treatment should be clinically guided and not necessarily as long as in reported cases in the literature.

## Conclusion

Empyema necessitans is a rare complication of pleuropneumonia in pediatrics. *A. actinomycetemcomitans* is a fastidious bacterium that requires a long culture period and antibiotic treatment. This case report details a case of pediatric empyema necessitans caused by *A. actinomycetemcomitans*, including histopathological findings, and provides a literature review of the few published cases in pediatrics. Our case highlights the importance of considering empyema necessitans in cases of a chest mass, even when malignancy seems the most probable diagnosis. It also emphasizes the need for a multidisciplinary approach before surgery. Surgical specimens should undergo culture as well as histopathological analysis, even if malignancy is suspected. Cultures must be performed on appropriate media and maintained for a sufficient length of time to identify fastidious organisms. Our case also suggests that treatment duration should be guided by clinical and paraclinical evolution in order to avoid overtreatment.

## Data Availability

The original contributions presented in the study are included in the article/Supplementary Material, further inquiries can be directed to the corresponding authors.

## Footnotes

^1^Columbia gelose

^2^Chocolate agar supplemented with PolyViteX
